# Fexofenadine Protects Against Intervertebral Disc Degeneration Through TNF Signaling

**DOI:** 10.3389/fcell.2021.687024

**Published:** 2021-08-24

**Authors:** Kaiwen Liu, Jianlu Wei, Guohua Li, Ronghan Liu, Dawang Zhao, Yuanqiang Zhang, Jie Shi, Qing Xie, Lei Cheng

**Affiliations:** ^1^Department of Orthopaedic Surgery, Qilu Hospital, Cheeloo College of Medicine, Shandong University, Jinan, China; ^2^School of Clinical Medicine, Shandong University, Jinan, China; ^3^Department of Orthopaedic Surgery, The Third Hospital of Hebei Medical University, Hebei, China; ^4^Shanxian Central Hospital, Shandong, China; ^5^Department of Orthopaedic Surgery, Jinan Central Hospital Affiliated to Shandong University, Jinan, China; ^6^Department of Pharmacy, Qilu Hospital, Cheeloo College of Medicine, Shandong University, Jinan, China

**Keywords:** intervertebral disc degeneration, fexofenadine, TNF-α, inflammation, CPLA2

## Abstract

**Objective:** Fexofenadine (FFD) is an antihistamine drug with an anti-inflammatory effect. The intervertebral disc (IVD) degeneration process is involved in inflammation in which tumor necrosis factor-α (TNF-α) plays an important role. This study aims to investigate the role of FFD in the pathological process of IVD degeneration.

**Methods:** Safranin O staining was used for the measurement of cartilageous tissue in the disc. Hematoxylin-Eosin (H&E) staining was used to determine the disc construction. A rat needle puncture model was taken advantage of to examine the role of FFD in disc degeneration *in vivo*. Western Blotting assay, immunochemistry, and immunoflurence staining were used for the determination of inflammatory molecules. ELISA assay was performed to detect the release of inflammatory cytokines. A real-time PCR assay was analyzed to determine the transcriptional expressions of molecules.

**Results:** Elevated TNF-α resulted in inflammatory disc degeneration, while FFD protected against TNF-α-induced IVD degeneration. Mechanism study found FFD exhibited a disc protective effect through at least two pathways. (a) FFD inhibited TNF-α-mediated extracellular matrix (ECM) degradation and (b) FFD rescued TNF-α induced inflammation in disc degeneration. Furthermore, the present study found that FFD suppressed TNF-α mediated disc degeneration via the cPLA2/NF-κB signaling pathway.

**Conclusions:** FFD provided another alternative for treating disc degeneration through a novel mechanism. Additionally, FFD may also be a potential target for the treatment of other inflammatory-related diseases, including IVD degeneration.

## Introduction

Intervertebral disc (IVD) degeneration is commonly prevalent in aging people and is the leading cause of low back pain (GBD 2016 Disease and Injury Incidence and Prevalence Collaborators, 2018). Despite the effectiveness of discectomy for IVD degenerative diseases, there is no identical drug for curing the degenerative pathological process (Cheng et al., 2018). IVD is the largest non-vascular tissue in the body and is composed of nucleus pulposus (NP), annulus fibrosus (AF), and cartilaginous endplates (CEPs). NP is a gelatinous matrix mainly composed of type II collagen (Col II) and proteoglycan with high water content. AF is divided into the outer and inner annulus. The extracellular matrix (ECM) of the outer annulus was mainly typed I collagen (Col I). The inner annulus is mainly fibrocartilage, which is composed of Col I and Col II and has a high content of proteoglycan (Blanquer et al., 2015). IVD degeneration is characterized by the destruction of the matrix and inflamed microenvironment, resulting in tissue breakdown. Importantly, inflammation is crucial for triggering IVD degeneration (Dudek et al., 2017; Alvarez-Garcia et al., 2018; Wang et al., 2018). Moreover, anti-inflammatory drugs achieve an encouraging outcome in treating IVD degeneration. However, the treatment for this disease is still demanding (Kamali et al., 2021).

Tumor necrosis factor-α (TNF-α) is one of the molecules in the TNF family (Croft et al., 2013; Wei et al., 2020a). TNF-α is regarded as the most powerful pro-inflammatory cytokine owing to the top position of the inflammatory cascade reaction (Tang et al., 2011; Liu and Bosch, 2012; Wei et al., 2018). It is well-accepted that the level of TNF-α is elevated in patients with IVD degeneration (Zhang et al., 2018; Yang et al., 2020). Additionally, the severity of IVD degenerative disease is closely associated with up-regulated TNF-α (Risbud and Shapiro, 2014). Interestingly, anti-TNF-α drugs have the potential for the prevention and treatment of IVD degeneration (Ding et al., 2017). Moreover, TNF-α exhibits its role mainly through two cell surface receptors, namely tumor necrosis factor receptor 1 (TNFR1) and tumor necrosis factor receptor 2 (TNFR2; Liu and Bosch, 2012; Zhao et al., 2015; Jian et al., 2018). Many anti-inflammatory drugs that inhibit TNF-α-induced catabolism also depend on TNFR1 signaling. All these findings prompt us to determine whether there is a new alternative target for treating IVD degeneration through the TNF-α pathway (Wei et al., 2016).

Fexofenadine (FFD) is an already discovered antihistamine drug with a highly selective HI receptor blocker and is commonly used in many allergic diseases, such as chronic idiopathic urticaria and rhinallergosis (Finn et al., 1999; Gelfand et al., 2003; Leurs et al., 2011). Among them, FFD can suppress eosinophilia and systemic anaphylaxis, and block the release of the inflammatory cytokines induced by the H1R/p38 and NF-κB pathway by blocking HI receptors (Watanabe et al., 2004; Park et al., 2014). Recent studies have found that FFD is a novel inhibitor of TNF/NF-κB signaling. Importantly, FFD is therapeutic in inflammatory arthritis by inhibiting TNF-α signaling (Liu et al., 2019).

Given that inflammation plays a key role in IVD degeneration and FFD has a strong anti-inflammatory effect, the purpose of this study was to investigate the role of FFD in IVD degeneration. The present study determined the treatment effect of FFD in the degenerative disc. Furthermore, the potential molecular mechanism involved is also illustrated.

## Materials and Methods

### Media, Regents, and Animal Models

Dulbecco’s Modified Eagle Medium Nutrient Mixture F-12 (DMEM/F-12) (catalog#8120482) was obtained from Gibco-BRL (Waltham, MA, United States). The following specific antibodies were used: anti-NOS-2 (Catalog.#AF0199), anti-MMP-13 (Catalog.#AF5355), anti-TNF Receptor I (Catalog.#AF0282), anti-Aggrecan (Catalog.#DF7561), and anti-GAPDH (Catalog.# AF7021) from Affinity Biosciences (OH, United States); anti-cPLA2 (catalog#YP0868), anti-p-cPLA2 (catalog#YT1084) from ImmunoWay Biotechnology (TX, United States); lamin B1 (catalog#13435), anti-P38 (catalog#8690), anti-p-P38 (catalog#4511), anti-ERK1/2 (catalog#9194), anti-p-ERK1/2 (catalog#4370), anti-p-P65 (catalog#3033), and anti-P65 (catalog#8242) from Cell Signaling Technology (Danvers, MA, United States); anti-COX-2 (catalog#ab179800) from Abcam (Cambridge, United Kingdom).

The mice and SD rats used in the experiment were purchased from the Animal Center of Shandong University. All of the animals were 8–12 weeks old male mice and rats, living in the same specific pathogen-free (SPF) standard environmental conditions (25°C, light/dark cycle for 12 h) with free access to food, and allowed to move freely. All experiments involving animals were carried out in accordance with the guidelines of the Professional Committee of Animal Care and Use of Shandong University (Shandong, China), which approved this study.

### Human Samples and Ethics Statement

This study was approved by the Medical Ethical Committee of Qilu Hospital of Shandong University. All the patients who participated in the study signed an informed consent document and voluntarily agreed to participate in this study. Human NP tissues were obtained from 40 patients who underwent lumbar spine surgery in Qilu Hospital, Shandong University. Human discs were collected from the operation of discectomy. In detail, the disc of Lumbar 4/5 was selected based on magnetic resonance imaging (MRI) scanning with Pfirrmann grade II. Specifically, Pfirrmann classification was divided into five grades (I–V) (Pfirrmann et al., 2001), This system is based on T2-weighted MRI, which essentially assesses water content based on signal intensity (Table 1). In total, 40 discs were used in the present study. Human NP tissues were isolated and stimulated with TNF-α and FFD for further experiments.

**TABLE 1:** Pfirrmann classification.

Grade	Structure	Distinction of NP and AF	Signal Intensity	Height of Disc
I	Homogeneous, bright white	Clear	Hyperintense or isointense to cerebrospinal fluid	Normal
II	Inhomogeneous, with or without horizontal bands	Clear	Hyperintense or isointense to cerebrospinal fluid	Normal
III	Inhomogeneous, gray	Unclear	Intermediate	Normal to slightly decreased
IV	Inhomogeneous, gray or black	Lost	Intermediate to hypointense	Normal to moderately decreased
V	Inhomogeneous, black	Lost	Hypointense	Collapsed disc space

### Isolation and Culture of Complete Mouse IVDs

After the mice were sacrificed, and the whole spine was extracted after confirming the anatomical position of the lumbar spine. The whole lumbar vertebrae were completely immersed in sterile phosphate buffered saline (PBS) containing 100 U/mL penicillin/streptomycin for 30 min. Then the lumbar vertebrae were washed with aseptic PBS three times. The lumbar tissue is then cut to an appropriate length and the integrity of the IVD was maintained. Finally, the mouse IVDs were carefully placed in six-well plates and cultured in DMEM/F-12 medium (Gibco, United States), which contained 10% fetal bovine serum (FBS, Gibco, United States), 1% 100 U/mL penicillin, and 100 mg/mL streptomycin (HyClone, United States), TNF-α(50 ng/mL, Abcam, United States) were added, with or without FFD treatment for 7 days, the medium was replaced every 3 days (Johnson et al., 2016).

### Needle Puncture Model of Rat

The needle puncture model of Rat was established according to the methods outlined literature. After the mice were sacrificed, the skin surface was disinfected with 75% alcohol. The area between the eighth and ninth coccygeal vertebrae (Co8-Co9) of the rats was punctured with a 20-gauge needle parallel to the caudal vertebrae, with a depth of 5 mm. The needle was then rotated 360° for 5 s. To eliminate the effects of injection, the same volume of PBS or FFD (10 mg/kg body weight) was injected intraperitoneally every other day (Liu et al., 2017; Tang et al., 2018).

### Histological Staining

The IVDs of mice and rats were fixed in 4% paraformaldehyde at room temperature. After 24 h, the samples were washed with PBS three times and decalcified by 10% EDTA solution (pH 7.2–7.4) within 2–3 weeks at room temperature. Decalcified tissue was embedded into paraffin blocks and cut into continuous tissue sections with a thickness of 5 μm. Tissue sections were stained with Safranin-O staining kit (G1371, Solarbio) and hematoxylin-eosin (H&E) staining kit (EE0012, Sparkjade) according to the procedure recommended by the manufacturer (Wei et al., 2017).

### Histological Assessment

The histological score was determined as previously described based on the H&E staining or Safranin O staining (Han et al., 2008). Briefly, cellularity and morphology of the AF, NP, and the border between the AF and NP was determined in this grading system. Histological score analysis included five categories. Total grades ranged from 5 to 15, in which normal disc get one point in each category and most severe degeneration scored 15. we determined the percentage of positive cells to analyze immunohistochemistry staining.

### Culture of Murine NP Cells and Primary Human NP Cells

In this study, the mice were killed by cervical vertebra dislocation and soaked in 75% ethanol for 20 min. Peel off the back and separate the entire spine. Separate the IVD, cut the IVD tissue into pieces, digest NP tissue with 0.25% trypsin (Gibco, United States) for 20 min, and then digest it with 0.2% collagenase (Sigma-Aldrich, St. Louis, MO, United States) for 4 h. The disc tissue was placed in a culture flask containing DMEM/F-12 medium (Gibco, United States), and adding 10% fetal bovine serum (FBS, Gibco, United States), 1% 100 U/mL penicillin, and 100 mg/mL streptomycin (HyClone, United States). All the flasks were cultured in an incubator with 37°C, 5% CO_2_, and 95% air. When the NP cells reached adhesion, the medium was changed. After that, the culture medium was changed every 2 days and passaged when the cells reached 80–90% cell density.

Human IVD tissues were collected from patients undergoing lumbar surgery. After rinsing with aseptic PBS three times, the CEPs and AF were removed. The rest of the processing steps were the same as above. In this study, we used second or third generation cells for all experiments.

### Real-Time Reverse Transcriptase-Polymerase Chain Reaction (RT-PCR)

Total RNA was extracted separately with TRIzol reagent (Takara Bio, Japan) from the NP tissues and NP cells of each indicated group. Complementary DNA (cDNA) was synthesized using an real-time reverse transcriptase-polymerase chain reaction (RT-PCR) kit (Toyota, japan) according to the manufacturer’s instructions. RT-PCR reaction was performed with an SYBR-Green-PCR Master Mix (Toyota, Japan) on a Bio-Rad CFX 96 system (Bio-Rad, CA, United States). The mRNA expression level of the related gene was calculated by the ΔΔCT method and the results were statistically analyzed. Matrix metalloproteinase-13 (MMP-13), Cyclooxygenase-2 (COX-2), Nitric oxide synthase-2 (NOS-2), Interleukin-6 (IL-6), Interleukin-1β (IL-1β), Interleukin-17 (IL-17), SOX-9, Aggrecan, Col-II, Bax, Caspase-3, Bcl-2, β-actin, and GAPDH nucleotide sequence specific primers are shown in Table 2.

**TABLE 2:** Primers used for quantitative real-time PCR.

Target	Forward primers, 5′–3′	Reverse primers, 5′–3′
*Human*		
MMP-13	ATTAAGGAGCATGGCGACTTCT	GCCCAGGAGGAAAAGCATGA
COX-2	TCAGCCATACAGCAAATCCTTG	GTCCGGGTACAATCGCGACTT
NOS-2	CGTGGAGACGGGAAAGAAGT	FACCCCAGGCAAGATTTGGA
IL-6	AGACAGCCACTCACCTCTTCA	GGCTTGTTCCTCACTACTCTC
IL-1β	GCCATGGACAAGCTGAGGAAG	GTGCTGATGTACCAGTTGGG
IL-17	CTGTCCCCATCCAGCAAGAG	AGGCCACATGGTGGAGAATC
Aggrecan	GGTCTCACTGCCCAACTACC	CACGATGCCTTTCACCACGA
COL-II	GATGGCTGCACGAAACATACC	GCCCTATGTCCACACCGAAT
SOX-9	AAGGACCACCCGGATTACAAG	GTTGGGGGAGATGTGCGTC
Bcl-2	TGTGGTCCATCTGACCCTCC	ACATCTCCCTGTTGACGCTCT
Bax	CTGAGCTGACCTTGGAGC	GACTCCAGCCACAAAGATG
Casp3	AGGAGGGACGAACACGTCT	CAAAGAAGGTTGCCCCAATCT
GAPDH	GCACCGTCAAGGCTGAGAAC	TGGTGAAGACGCCAGTGGA
*Mouse*		
MMP-13	TGATGATGAAACCTGGACAAGCA	GGTCCTTGGAGTGATCCAGACCTA
COX-2	GCATTCTTTGCCCAGCACTT	ACCTCTCCACCAATGACCTGA
NOS-2	CCTGCTTTGTGCGAAGTGTC	CCCAAACACCAAGCTCATGC
IL-6	GCCTTCTTGGGACTGATGCT	GCCATTGCACAACTCTTTTCTCA
IL-1β	GTGTCTTTCCCGTGGACCTT	AATGGGAACGTCACACACCA
IL-17	GGAGAGCTTCATCTGTGTCTCTG	TTGGCCTCAGTGTTTGGACA
Aggrecan	AAACCTGGCGTGAGAACTGT	CCACTGACACACCTCGGAAG
COL-II	CCAGATTGAGAGCATCCGCA	ACTTTCATGGCGTCCAAGGT
SOX-9	CACAAGAAAGACCACCCCGA	CTCCGCTTGTCCGTTCTTCA
β-action	GGCTGTATTCCCCTCCATCG	CCAGTTGGTAACAATGCCATGT
*Rat*		
Aggrecan	CTGAATGGGAGCCAGCCTAC	GATGTGGAAGGGACTTGCGA
COL-II	CAAAGGTGCTCGAGGAGACA	AGAACCAGAGGGACCGTCAT
β-action	CTCTGTGTGGATTGGTGGCT	CGCAGCTCAGTAACAGTCCG

### Isolation and Culture of Human IVD Tissues and Immunohistochemistry

Human IVD tissue comes from patients undergoing lumbar surgery. After removing the fibrous annulus and cartilage endplate, the NP tissue was cultured in DMEM/F-12 medium (Gibco, United States) supplemented with 10% fetal bovine serum (FBS, Gibco, United States), 1% 100 U/mL penicillin, and 100 mg/mL streptomycin (HyClone, United States).

The NP tissue was cultured in a complete medium containing TNF-α (10 ng/mL, Abcam, United States) in a six-hole plate for 7 days, and the medium was replaced every 2 days in the absence or presence of FFD. It was then fixed with 4% paraformaldehyde for 72 h and embedded in paraffin tissue. The tissue was cut into 5 μm thick sections, then dewaxed with xylene and treated with ethanol gradient, and then the antigen was repaired with 0.125% trypsin (ZSGB-Bio, Beijing, China) for 30 min. It was incubated with 3% hydrogen peroxide then the non-specific binding site of the tissue was closed with 20% goat serum (ZSGB-Bio, Beijing, China) for 20 min. The sections were incubated overnight with rabbit anti-human NOS-2 (1:200, Affinity Biosciences, United States), rabbit anti-human MMP-13 (1:200, Affinity Biosciences, United States), rabbit anti-human COX-2 (1:200, Abcam, United States), and rabbit anti-human Aggrecan (1:50, Affinity Biosciences, United States) under 4°C conditions. Then sections were incubated at room temperature with goat anti-rabbit immunoglobulin (IgG)-horseradish peroxidase (HRP) secondary antibody (1:200, ZSGB-Bio, China) for 1 h. The results were quantified using the Image-Pro Plus 6.0 software (Media Cybernetics, Inc., United States).

### Western Blotting

Human and mouse NP tissues cultured *ex vivo*, NP tissues isolated from rat, as well as human and mouse NP cells treated in different experimental conditions, were treated with RIPA buffer (P0013C, Beyotime Biotechnology) containing PMSF (ST506, Beyotime Biotechnology) and Protein Phosphatase Inhibitor (P1260, Solarbio) and incubated on ice for 40 min. The total protein lysate was transferred into a 1.5 ml centrifuge tube and centrifuged at 12,000 rpm for 15 min under 4°C conditions. The cytoplasmic proteins and nucleoproteins were extracted using a nucleoprotein extraction kit (P0027, Beyotime Biotechnology) to carefully absorb the supernatant and determine the protein concentration with BCA Protein Assay Kit (Beijing Biotechnology Co., Beijing, China). After calculation, equal amounts of each group of proteins were separated in 10% SDS-PAGE and then transferred to the PVDF membrane (Millipore, United States). The Tris buffer saline Tween-20 (TBST) containing 5% BSA was used to block PVDF membrane for 1 h at room temperature, and then incubated overnight under 4°C conditions with first-order antibodies (rabbit anti-MMP-13, NOS-2, GAPDH, 1: 1,000, Affinity Biosciences, United States; rabbit anti-COX-2, 1:1,000, Abcam, United States; rabbit anti-p38, p-P38, p65, p-P65, Erk1/2, p-ERK1/2, 1;1000, Cell Signaling Technology, United States; rabbit anti- cPLA2, P-cPLA2,1:1000, ImmunoWay Biotechnology, United States). We then washed the membrane with TBST three times for 10 min each time. The PVDF membrane was cultured at room temperature in goat anti-rabbit IgG-HRP secondary antibody (1:3000; Beijing Golden Bridge Biotechnology, China) for 1 h. The bound antibody was visualized using an enhanced chemiluminescence system (Amersham Life Science, Little Chalfont, United Kingdom).

### ELISA

Human NP cells were incubated with TNF-α (10 ng/ml), Arochidonic Acid (10 μM) and different concentration gradient FFD (1 μM, 10 μM) for 48 h, then the cell culture supernatant was collected. The FFD treatment group cells were pre-incubated with different concentration gradient FFD overnight. Next, the cytokine secretory expression levels of IL-1β, IL-6, IL-17, and AA were measured by ELISA kit (Elabscience, United States).

### Immunofluorescence Staining

Cells were fixed with 4% formaldehyde for 10 min and permeabilized in 0.2% Triton-X for 15 min, then block 1% BSA at room temperature for 30 min. The cells were then incubated with rabbit anti-human p65 (1:400, Cell Signaling Technology, United States) first antibody overnight at 4°C. On the second day, the cells were washed with PBS, and then incubated at room temperature for 1 h with secondary fluorescence labeled sheep anti-rabbit IgG antibody (1:200 ZSGB-BIO, China). The image was taken by a fluorescent microscope (Olympus IX51, Japan).

### TUNEL Staining

To test whether FFD affected the apoptosis of human NP cells, cells were seeded in a 24-well plate. FFD (10 μM) was used for the pre incubation overnight, followed by TNF-α stimulation for 24 h. Cells were stained with TUNEL Assay Kit (E-CK-A320; Elabscience Biotechnology). All procedures were performed according to the manufacturer’s instructions.

### Statistical Analysis

Total data acquisition was conducted in a blinded manner. For comparison of various treatment groups, the unpaired Mann–Whitney *t*-test, paired Student’s *t*-test, and 1-way or 2-way ANOVA (when appropriate) were performed. The experimental data were statistically analyzed using GraphPad prism 7. All data were mean ± standard deviation (SD). *p* < 0.05 was considered statistically significant.

## Results

### FFD Protected Against TNF-α-Induced Intervertebral Disc Degeneration

**FIGURE 1:**
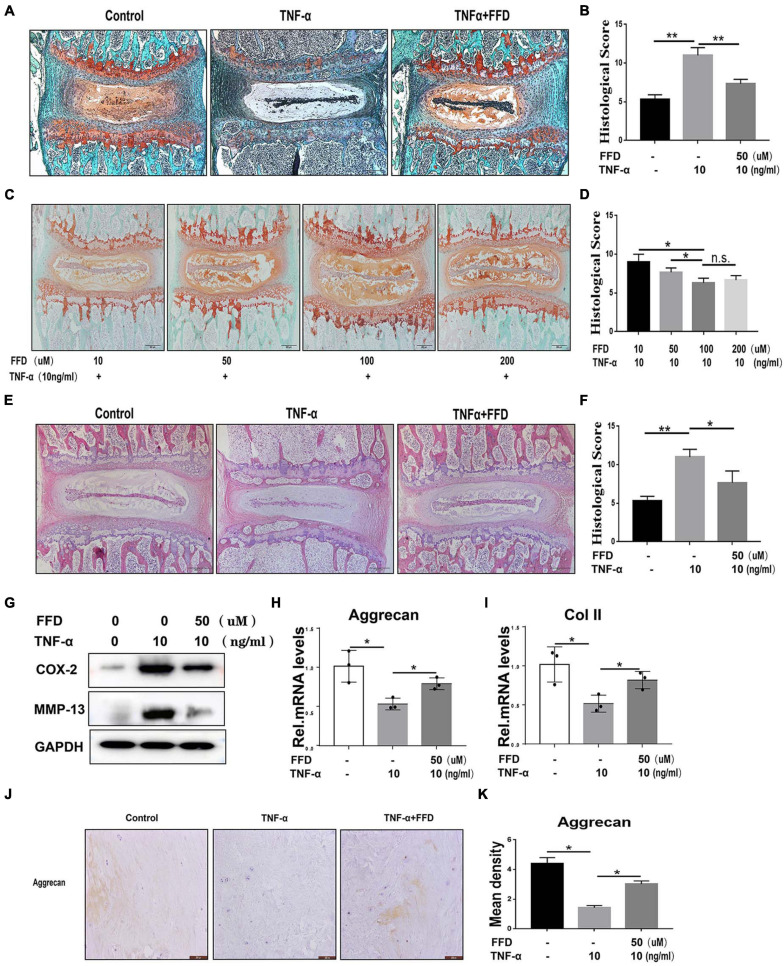
Fexofenadine (FFD) protected against tumor necrosis factor-α (TNF-α)-induced intervertebral disc degeneration. **(A)** Safranin O staining of mouse disc. The discs were cultured and treated with TNF-α (10 ng/ml) in the presence or absence of FFD (50 μM) for 7 days. **(B)** Histological score analysis based on the Safranin O staining (*n* = 6). Scale bar = 200 μm. **(C)** The mouse disc were cultured in presence of FFD with various concentration conditions (10, 50, 100, and 200 μM), the effect of the drug was determined by Safranin O staining (*n* = 6). **(D)** Histological score based on the Safranin O staining. Scale bar = 200 μm. **(E)** H&E staining of mouse disc. The mouse discs were cultured in medium supplemented with 10 ng/ml TNF-α in the absence or presence of FFD (50 uM) for 7 days. **(F)** Histological score of mouse discs in each indicated group (*n* = 6). Scale bar = 200 μm. **(G)** Protein was extracted from mouse discs, Western Blotting analysis of COX-2 and MMP-13 was determined. **(H,I)** Transcriptional level of Aggrecan and Col II from mouse discs in each group. Each experiment was performed three times. **(J)** Immunohistochemistry staining of Aggrecan in human disc organ. The human discs were treated without or with TNF-α (10 ng/mL) in the absence or presence of FFD (50 μM) for 7 days (*n* = 6). Brown signal indicates positive. Scale bar = 200 μm. **(K)** Statistical analysis of the percentage of the positive cells based on immunohistochemistry staining. The values shown represent the mean ± standard deviation *p < 0.05 and **p < 0.01 vs. control group.

To determine whether FFD inhibits TNF-α-mediated disc degeneration, we took advantage of mouse disc by *ex vito* culture. The primary mouse IVDs were cultured with or without 10 ng/ml TNF-α in the presence or absence of 50 uM FFD for 7 days. As shown in Figure 1A, Safranin O staining indicated TNF-α mediated remarkable loss of cartilage tissue. However, additional use of FFD effectively rescued TNF-α-caused cartilagous tissue destruction. Moreover, based on the histological score (Figure 1B), FFD significantly reduced TNF-α-induced caritilagous tissue loss. Furthermore, to investigate the optimum concentration of FFD in disc degeneration, primary mouse discs were cultured with TNF-α with a concentration of 10 ng/ml in the presence of FFD with various concentrations. As illustrated in Figures 1C,D, FFD effectively inhibited TNF-α-mediated disc destruction. To further examine whether FFD protects the whole tissue structure, H&E staining was taken. As indicated in Figures 1E,F, TNF-α caused disc deteriorative change. In contrast, additional use of FFD effectively restored TNF-α-mediated destruction. To further examine the effect of FFD in disc degeneration, we extracted the tissues and followed them by the protein test. As indicated in Figure 1G, the protein level of pro-inflammatory molecules, such as COX-2 and MMP-13, was increased in the presence of TNF-α, while FFD reduced TNF-α-induced the expression of these molecules. Importantly, to examine the level of matrix content change, we extracted the mRNA from tissues, which was followed by a Real-time PCR assay. As shown in Figures 1H,I, FFD significantly reversed TNF-α-mediated loss of matrix content. To further determine the matrix content expression of Aggrecan, we cultured human disc in the presence of TNF-α with or without FFD. As illustrated in Figures 1J,K, immunohistochemistry staining of Aggrecan was taken to confirm FFD-mediated matrix content improvement in human tissues. Collectively, FFD effectively protected against IVD degeneration by restoring cartilagous tissues and remaining the disc structure.

### FFD Inhibited Intervertebral Disc Degeneration in the Tail Injury Rat Model

**FIGURE 2:**
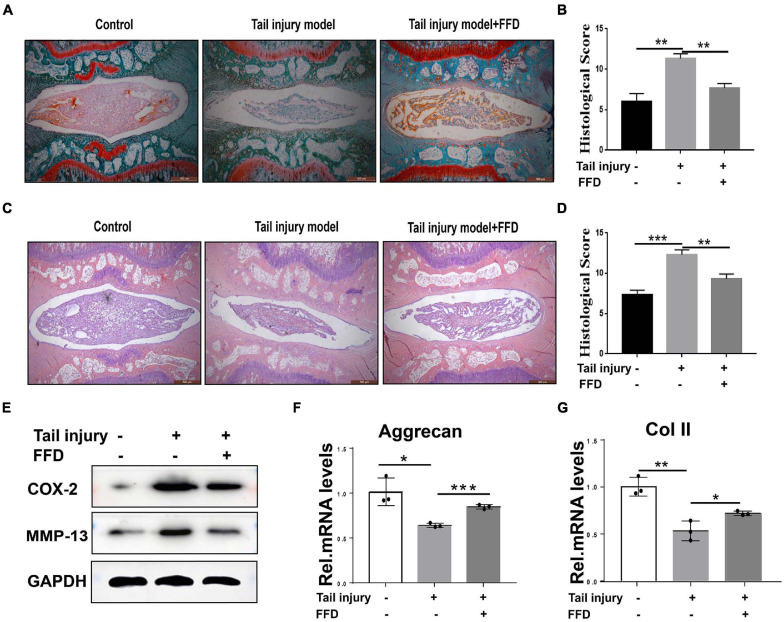
Fexofenadine (FFD) inhibited intervertebral disc degeneration in rat tail injury model. **(A)** Safranin O staining of rat disc. The tail injury model was established in 8-week-old rat (*n* = 6). **(B)** Histological score analysis based on the Safranin O staining. Scale bar = 500 μm. **(C)** H&E staining of tail Injury model (*n* = 6). **(D)** Histological score based on H&E staining. Each indicated group contained 6 rat discs. Scale bar = 500 μm. **(E)** Western blotting assay determined the protein level of COX-2 and MMP-13 in the rat disc injury model. **(F,G)** The transcriptional expressions of Aggrecan and Col II from rat tail injury model were detected by Real-time PCR assay. Each experiment was performed three times independently. The values shown represent the mean ± standard deviation.*p < 0.05, **p < 0.01, and ***p < 0.001 vs. control group.

To further determine whether FFD attenuate degenerated discs, we took advantage of the tail injury rat model. After the disc was degenerated by needle punch, FFD was systematically delivered. As illustrated in Figures 2A,B, Safranin O staining indicated cartilagous tissue was remarkably lost after the model was established. However, FFD effectively reversed this situation. Additionally, density analysis suggested FFD significantly prevented cartilage loss. Moreover, as shown in Figures 2C,D, H&E staining indicated that the histological score was increased in the degenerated group while the FFD-treated group exhibited restoration of the disc. Furthermore, the protein was extracted from the disc tissues, as illustrated in Figure 2E, the expression of pro-inflammatory biomarkers, including COX-2 and MMP-13, were increased in the disc injury model, while FFD decreased these molecules’ expression. Additionally, as demonstrated in Figures 2F,G, the transcriptional level of matrix content, such as Aggrecan and Col II, were also rescued by FFD in the rat disc injury model. Above all, FFD effectively attenuated disc degeneration.

### FFD Inhibited TNF-α-Mediated Extracellular Matrix Degradation

**FIGURE 3:**
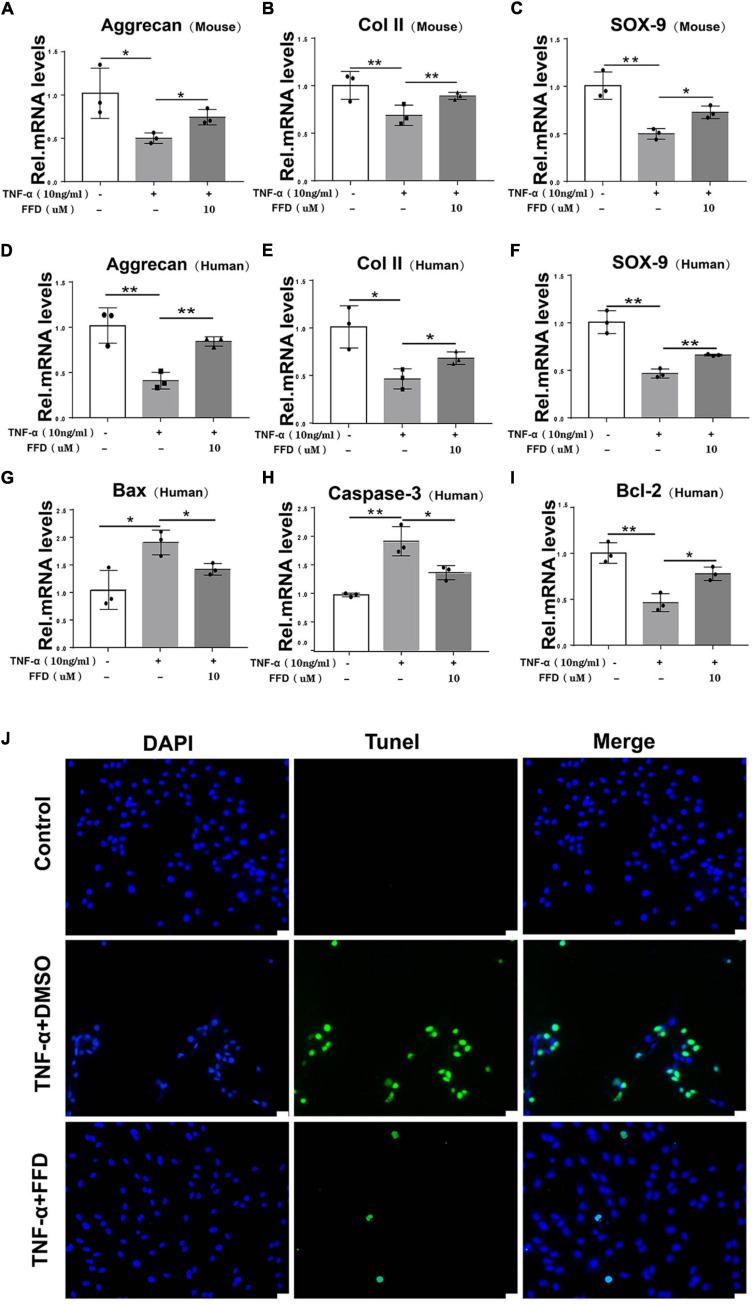
Fexofenadine (FFD) inhibited TNF-α-mediated ECM degradation and apoptosis in NP cells. **(A–C)** The mouse NP cell of P0 or P1 were treated with or without TNF-α (10 ng/mL) in the absence or presence of FFD (10 μM). mRNA expressions of Aggrecan, COL-II, and SOX-9 were tested by real-time PCR. Each experiment was performed three times independently. **(D–F)** The human NP cell of P0 or P1 were treated without or with TNF-α (10 ng/mL) in the absence or presence of FFD (10 μM). The mRNA expression levels of Aggrecan, COL-II, and SOX-9 were detected by Real-time PCR assay. Each experiment was performed three times independently. **(G–I)** The transcriptional levels of Bax, Caspase-3, and Bcl-2 in human NP cells. Each experiment was performed three times independently. **(J)** TUNEL staining of the human NP cells in each group. The human NP cells were cultured with TNF-α (10 ng/mL) in the absence or presence of FFD (10 μM) for 24 h. Scale bar = 100 μm. The values shown represent the mean ± standard deviation. *p < 0.05 and **p < 0.01 vs. control group.

The progressive destruction of the ECM is known to be the most important pathological feature of IVD degeneration. NP is a colloidal matrix consisting mainly of type II collagen and Aggrecan which maintains the whole construct. Next, we sought to determine the role of FFD for the ECM in disc degeneration. To address this issue, primary mouse NP cells were cultured with or without TNF-α in the absence or presence of FFD. As indicated in Figures 3A–C, TNF-α significantly reduced the transcriptional expression of Aggrecan, Col II, and Sox 9, while additional use of FFD significantly up-regulated their expressions. Importantly, to further investigate whether this is the case in human NP cells, we took advantage of primary NP cells and cultured these cells in P0 generation. As illustrated in Figures 3D–F, FFD rescued TNF-α-induced regulation of transcriptional expression of Aggrecan, Col II, and Sox 9. Importantly, to investigate the role of FFD in cell apoptosis, we took advantage of human primary NP cells. As suggested in Figures 3G–I, FFD significantly rescued TNF-α-induced transcriptional expression of Bax, Caspase-3, and Bcl-2. Additionally, as shown in Figure 3J, the TUNEL assay indicated that FFD remarkably reversed TNF-α-mediated cell apoptosis. Above all, FFD rescued TNF-α-induced matrix destruction in disc degeneration.

### FFD Inhibited TNF-α Induced Inflammation in Disc Degeneration

**FIGURE 4:**
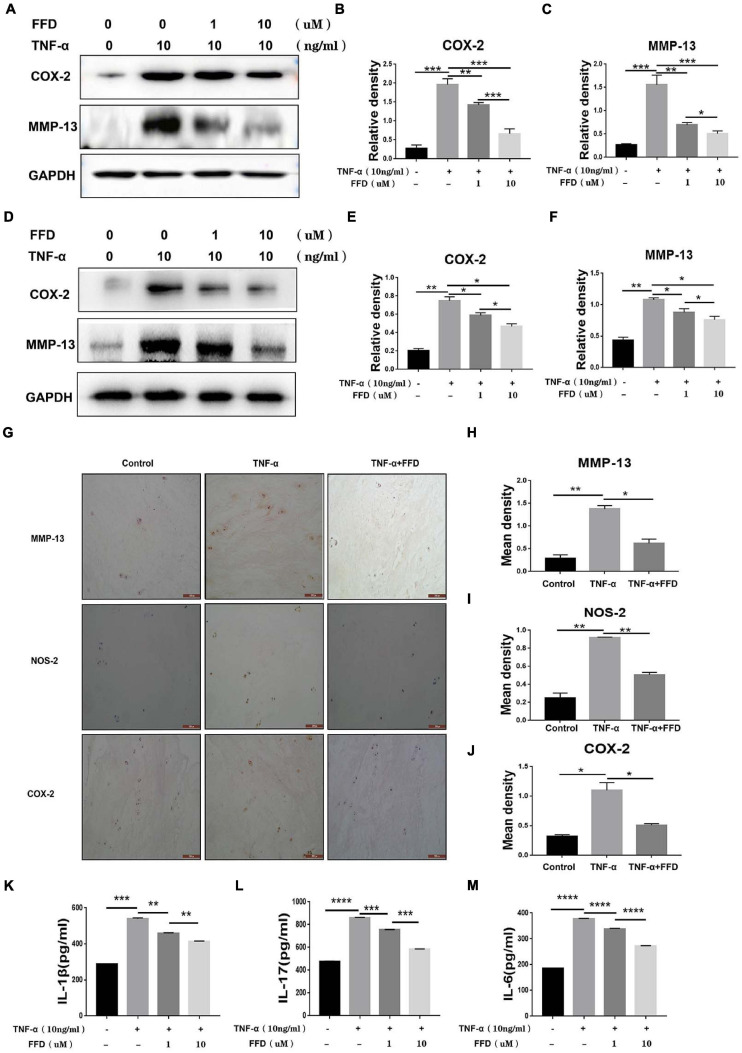
Fexofenadine (FFD) inhibited TNF-α induced inflammation in disc degeneration. **(A)** The protein expression of mouse COX-2 and MMP-13 were detected by Western blotting assay. The mouse NP cells were treated with TNF-α (10 ng/ml) with or without additional treatment with FFD (1 μM, 10 μM) for 48 h, followed by protein examination. **(B,C)** Quantification of the band density for mouse COX-2 and MMP-13 was based on the Western blotting assay. **(D)** The protein expression of human COX-2 and MMP-13 were detected by Western blotting assay. The human NP cells were treated with TNF-α (10 ng/ml) with or without additional treatment with FFD (1 μM, 10 μM) for 48 h, followed by protein examination. **(E,F)** Quantification of the band’s density for human COX-2 and MMP-13 was based on Western blotting assay. **(G)** Immunohistochemistry staining of MMP-13, NOS-2, and COX-2 in human discs. The human discs were treated without or with TNF-α (10 ng/mL) in the absence or presence of FFD (50 μM) for 7 days (*n* = 6). Scale bar = 200 μm. Brown signal indicated positive. **(H–J)** Quantification of the percentage of the positive cells was based on immunohistochemistry staining. **(K–M)** The expression of IL-1β, IL-6, and IL-17 in the culture media of each group, as detected by ELISA assay (*n* = 3). The human NP cells were treated without or with TNF-α (10 ng/mL) in the absence or presence of FFD (1 μM, 10 μM) for 48 h. Then the supernatant were collected for ELISA. The values shown represent the mean ± standard deviation. *p < 0.05, **p < 0.01, and ***p < 0.001 vs. control group.

Taking into account the importance of TNF-α in the inflammatory process in disc degeneration, we next investigated whether FFD exhibits its role by antagonizing TNF-α’s role. To address this issue, we cultured primary mouse NP cells and analyzed the expression of inflammatory molecules. As shown in Figures 4A–C, Western Blotting assay indicated the protein expression of proinflammatory molecules, including COX-2 and MMP-13, which were dramatically upregulated in the presence of TNF-α. However, FFD remarkably reduced these molecules’ expression in a dose-dependent manner. Human primary NP cells were also used to confirm this finding. As demonstrated in Figures 4D–F, FFD significantly decreased TNF-α-induced protein expressions of COX-2 and MMP-13. Furthermore, we collected primary human discs and cultured them with or without TNF-α in the absence or presence of additional FFD. As illustrated in Figure 4G, immunochemistry staining suggested TNF-α increased expression of COX-2, NOS-2, and MMP-13 in disc tissue. However, the presence of FFD significantly reduced the protein expression of these molecules in the disc tissue. Statistical analysis suggested the difference is significant (Figures 4H–J). Besides the mentioned pro-inflammatory molecules, we next determined whether the expression of TNF-α induced interleukin family cytokines was also altered. To attempt to figure it out, we cultured the primary human NP cells in conditional medium for 2 days. Supernate was collected and an ELISA assay was taken. As suggested in Figures 4K–M, TNF-α significantly increased cytokine expression of IL-1β, IL-17, and IL-6. Interestingly, additional use of FFD significantly decreased the expression of these cytokines.

**FIGURE 5:**
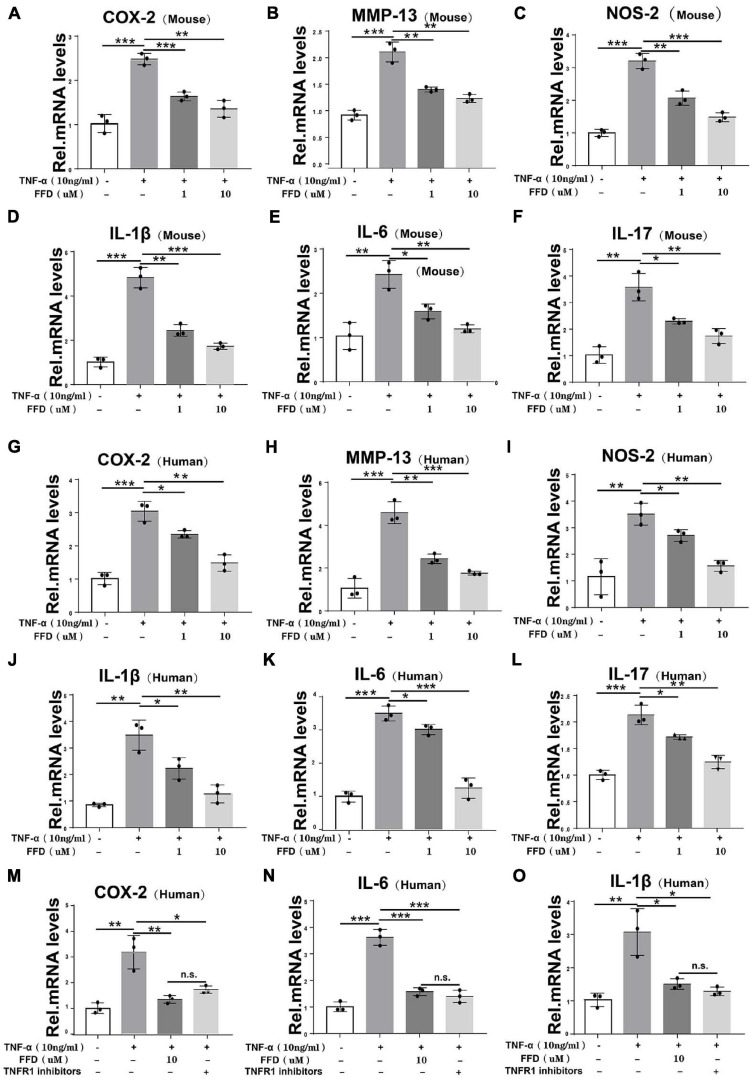
Fexofenadine (FFD) inhibited TNF-α induced inflammation in disc degeneration. **(A–F)** The transcriptional expression of COX-2, MMP-13, NOS-2, IL-1β, IL-6, and IL-17 in mouse NP cells, as measured by Real-time PCR assay. Each experiment was performed 3 times independently. **(G–L)** The transcriptional expression of COX-2, MMP-13, NOS-2, IL-1β, IL-6, and IL-17 in human NP cells, as determined by Real-time PCR assay. Each experiment was performed three times independently. **(M–O)** The transcriptional expression of COX-2, IL-6, and IL-1βin human NP cells, as determined by real-time PCR assay. The human cells were cultured with or without TNF-α (10 ng/mL) in presence of FFD or TNFR1 inhibitor. Each experiment was performed three times independently. Normalized values were calibrated against the control group and given a value of 1. *p < 0.05, **p < 0.01, and ***p < 0.001 vs. control group.

To further determine the involved mechanism, we cultured both mouse and human primary NP cells for 8 h and a Real-time PCR assay was taken. As indicated in Figures 5A–C, the TNF-α-upregulated transcriptional level of mouse COX-2, NOS-2, and MMP-13 was significantly decreased by FFD in the primary human NP cells. Additionally, as shown in Figures 5D–F, the transcriptional level of mouse interleukin family, such as IL-1β, IL-17, and IL-6, was also dramatically altered in the presence of FFD. This finding was also confirmed in the human NP cells as illustrated in Figures 5G–L. Since TNF-α mainly exhibited its inflammatory catabolic effect through TNFR1, herein, we compared FFD with TNFR1 inhibitor for the efficacy of blocking the TNF-α effects on disc degeneration. To address this issue, we isolated primary human NP cells and cultured them with TNF-α in the absence or presence of FFD. As indicated in Figures 5M–O, TNF-α up-regulated the transcriptional level of pro-inflammatory biomarkers, including COX-2, IL-6, and IL-1β. Additionally, both FFD and TNFR1 inhibitors significantly down regulated these molecules. Interestingly, the FFD and TNFR1 inhibitors did not show a big difference. FFD effectively protected against disc degeneration by antagonizing TNF-α-mediated destructive inflammation.

### FFD Suppressed TNF-α Mediated Disc Degeneration via NF-κB Signaling Pathway

**FIGURE 6:**
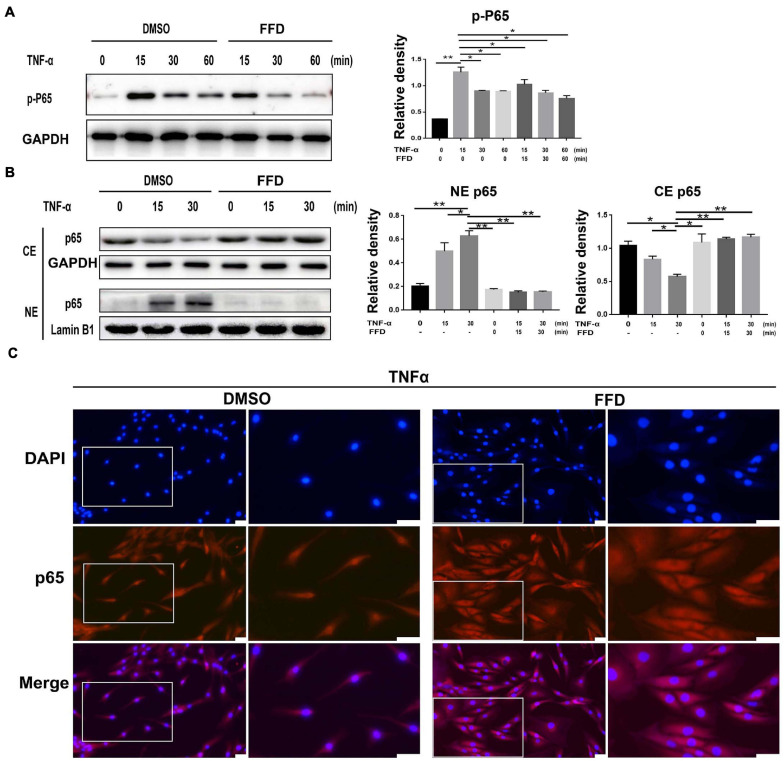
Fexofenadine (FFD) suppressed TNF-α mediated disc degeneration via NF-κB signaling pathway. **(A)** The human NP cell were treated without or with TNF-α (10 ng/mL) in absence or presence of FFD (10 μM), and collected at various time points, followed by Western blot analysis using image J (*n* = 3). **(B)** The human NP cell were treated with TNF-α (10 ng/mL) in the absence or presence of FFD (10 μM) for various time points, the cytoplasmic and nuclear protein fractions were extracted to test the nuclear translocation of p65 (*n* = 3). **(C)** The human NP cells were treated with or without FFD (10 μM) overnight followed by additional use of TNF-α (10 ng/mL) for 6 h. Immunofluorescence cell staining was performed to test the location of p65. Scale bar = 100 μm. The values shown represent the mean ± standard deviation. *p < 0.05 and **p < 0.01 vs. control group.

Tumor necrosis factor-α/nuclear factor-κB (NF-κB) signaling is a classical pathway. In the attempt of determining whether FFD altered TNF-α/NF-κB signaling, primary human NP cells were collected and cultured. As indicated in Figure 6A, FFD remarkably decreased the phosphorylation of P65 at indicated various time points, suggesting FFD inhibited TNF-α-induced NF-κB activation. Consequently, to examine whether FFD altered P65 translocation, the cytoplasm, and nuclear protein were extracted separately, followed by Western Blotting analysis. As shown in Figure 6B, the total expression of P65 in the cytoplasm decreased while that of P65 in nuclear was increased with time going by in the presence of TNF-α. However, the presence of FFD effectively inhibited the translocation of P65 from the cytoplasm into nuclear. To further confirm this finding, we cultured the primary NP cells and conducted immunofluorescence staining. As indicated in Figure 6C, TNF-α effectively activated and translocated P65 from the cytoplasm into nuclear. However, additional use of FFD largely abolished TNF-α mediated P65 translocation in the primary NP cells. FFD effectively inhibited TNF-α/NF-κB signaling in the process of disc degeneration.

### FFD Attenuated TNF-α-Mediated Disc Degeneration by Suppressing cPLA2

**FIGURE 7:**
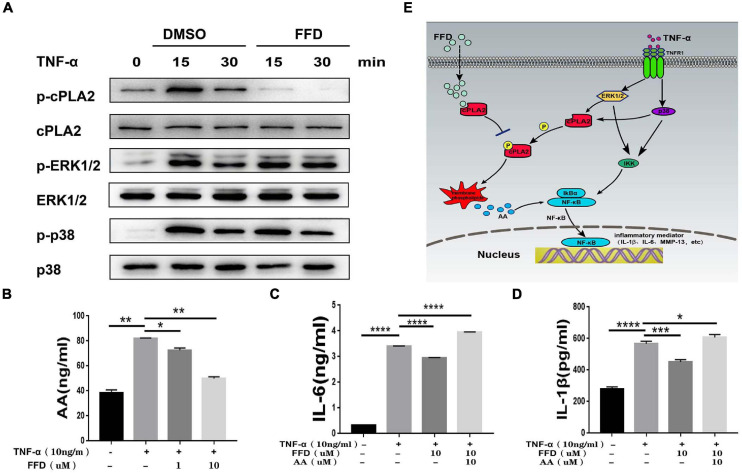
Fexofenadine (FFD) attenuated TNF-α-mediated disc degeneration by suppressing cPLA2. **(A)** The human NP cells were treated with TNF-α (10 ng/mL) in the absence or presence of FFD (10 μM) for various time points. p-p38, p38, p-ERK1/2, ERK1/2, p-cPLA2, and cPLA2 were detected by Western blot with corresponding antibodies. **(B)** The AA levels in NP cells without or with TNF-α (10 ng/mL) in absence or presence of FFD (1 μM, 10 μM) for 48 h were examined by ELISA (*n* = 3). **(C,D)** The NP cells were treated with TNF-α (10 ng/mL), AA (10 μM), and FFD (10 μM) as indicated. The levels of IL-6 and IL-1β were detected by ELISA (*n* = 3). The values shown represent the mean ± standard deviation. *p < 0.05 and **p < 0.01 vs. control group. **(E)** A proposed model for explaining the anti-TNF activity of FFD in intervertebral disc degeneration.

Besides classical TNF-α/NF-κB signaling, it is widely accepted that TNF-α activates MAPK and Erk1/2 signaling. To further determine whether FFD also altered TNF-α induced other signaling, we took advantage of primary NP cells. As illustrated in Figure 7A, FFD showed no alternative change for TNF-α induced Erk1/2 and P38 phosphorylation. Importantly, cPLA2 was found to be increased in the presence of TNF-α in many inflammatory diseases. Interestingly, a recent study found that FFD could bind to cPLA2, and remarkably decreased cPLA2 phosphorylation in NP cells. Consequently, activated cPLA2 promoted phospholipid hydrolysis to produce arachidonic acid (AA), followed by activation of NF-κB signaling. To confirm this finding, we detected the AA level of cell supernatant by ELISA kit. As shown in Figure 7B, FFD significantly inhibited TNF-α induced AA production in a dose-dependent manner. To confirm this finding, we next determined whether additional use of AA could reverse FFD alleviated TNF-α-mediated inflammation. To address this issue, we cultured the primary NP cells and collected the supernate of the medium. ELISA assay was taken to detect the protein expression of proinflammatory cytokines. As suggested in Figures 7C,D, the expression of L-1βand IL-6 was significantly increased in the presence of TNF-α, while FFD significantly decreased the expression of these molecules in the presence of TNF-α. Interestingly, the cytokine expression returned when AA was added with FFD. FFD attenuated disc degeneration also through the cPLA2 pathway.

## Discussion

Intervertebral disc-derived low back pain is a common cause of low back pain, with the aging of the population, the incidence is gradually increasing (GBD 2016 Disease and Injury Incidence and Prevalence Collaborators, 2018; Hartvigsen et al., 2018). IVD degeneration is a basic cause of low back pain and a common degenerative disease (Lyu et al., 2021). Unfortunately, there are currently no drugs to block the progression of IVD degeneration (Cheng et al., 2018), and end-stage patients have to undergo surgical treatment, but surgical treatment can aggravate the degeneration of adjacent segments of the spine and bring new problems (Okuda et al., 2018).

Despite the complex etiology of IVD degeneration, the involvement of inflammatory responses, like many other chronic diseases, is a common feature (Risbud and Shapiro, 2014; Dudek et al., 2017; Alvarez-Garcia et al., 2018). In the process of IVD degeneration, the expression of pro-inflammatory cytokines in IVD cells increases and leads to the degradation of the ECM, which leads to changes in the structure and biomechanics of the IVD. At the same time, the inflammatory response mediated by immune cells was enhanced in the degenerated IVD, which led to the decrease of the number of NP cells and the deterioration of the IVD microenvironment (Zhao et al., 2020; Lyu et al., 2021). TNF-α is at the top of the inflammatory cascade and plays an important role in the progression of IVD degeneration (Croft et al., 2013; Wei et al., 2020a). TNF-α is a powerful inflammatory factor that is positively correlated with the degree of IVD degeneration (Risbud and Shapiro, 2014; Zhang et al., 2018; Yang et al., 2020). Activation of the TNF-α signaling leads to pain in the IVD innervated by abundant nerves, and TNF-α expression levels were positively correlated with pain levels. IL-1β, IL-6 induced by TNF-α can participate in IVD degeneration by promoting matrix degradation and inhibiting synthesis. IL-1β leads to an increase in the expression of matrix degrading enzymes such as MMP-13 (Chen et al., 2020), which are the main proteases that directly disrupt IVD tissue (Wang et al., 2020). Based on this, more and more studies have been devoted to blocking TNF-α mediated inflammatory response to delay disc degeneration and achieve positive therapeutic effects.

Fexofenadine is a drug found to have anti-TNF-α effects in recent years. FFD is a safe H1 receptor blocker commonly used in the treatment of allergic rhinitis. FFD was selected from an FDA-approved drugs library, with better safety, the convenience of fewer side effects. This will save a large quantity of funding and research time. Unlike traditional anti-TNF-α drugs, FFD does not block the binding of TNF-α to receptors on the cell surface but specifically acts on the downstream key inflammatory factor cPLA2 (Liu et al., 2019).

cPLA2 contains three domains, including the Ca2 binding domain (C2D), catalytic domain 1 (CD1), and catalytic domain 2 (CD2; Clark et al., 1991). The study found that cPLA2 in bone marrow derived macrophage (BMDM) cells was under TNF-α action and can be activated by phosphorylated Erk1/2 and p38. FFD can target and directly bind to site Ser-505 and inhibit its phosphorylation by Erk1/2 and p38, which in turn inhibits cPLA2 activity, the production of AA, and the production of inflammatory factors mediated by NF-κB (Liu et al., 2019). Based on the important role of TNF-α in IVD degeneration and the anti-inflammatory effect of FFD in previous studies, the present study determined the role of FFD in disc degeneration.

To verify the therapeutic effect of FFD in IVD degeneration, we first used mouse IVD for *in vitro* study and found that TNF-α accelerated cartilage loss. There was no blood supply in the mature IVD, which is considered the largest non-vascular tissue in the human body. Its nutrition metabolism functions mainly through cartilage endplate diffusion, fiber ring diffusion, and other ways. The degeneration of the cartilage endplate destroyed the homeostasis of the disc and accelerated the degeneration of the IVD (Jiang et al., 2019). The present study found that FFD protected against cartilage degeneration and exhibited therapeutic effects in a rat model.

It is generally accepted that TNF-α is a proinflammatory cytokine that also induces other proinflammatory molecules, such as IL-1β, MMP-13, COX-2, and NOS-2 (Vanamee and Faustman, 2018). There were two related receptors on the cell surface, namely TNFR1 and TNFR2. TNFR1 is thought to mediate the inflammatory responses (Ding et al., 2021). In detail, TNF-α binded to TNFR1, followed by activation of p38 and ERK1/2 (Wei et al., 2020b), the phosphorylation of cPLA2 further occurred. After that, AA production increased, and the increased AA activated NF-κB signaling (Korotkova and Lundberg, 2014). NF-κB was phosphorylated and the subunit of P65 shifted into the nucleus, leading to the production of down-stream inflammatory molecules (Figure 7E). The mature NP was rich in proteoglycans and type II collagen, and the increase of MMP-13 expression accelerated the degradation of the IVD matrix, including proteoglycan and type II collagen. In line with previous publications, our results showed that FFD significantly decreased the expression of the matrix-destructive cytokines. Unlike previous anti-TNF-α drugs, FFD showed no alteration for phosphorylation of p38 or ERK1/2. Interestingly, FFD largely inhibited the phosphorylation of cPLA2. FFD spread directly into the cell without binding to the cell surface, followed by blocking of the cPLA2/AA/NF-κB inflammatory factor production pathway (Liu et al., 2019).

It is accepted that TNF-α is elevated in the process of disc degeneration both systemically or locally. Previous and present studies have indicated that FFD could inhibit TNF-α-induced disc degeneration. However, whether this FFD mediated the disc-protective effect through antagonizing the systemic or local TNF-α’s effect remains unclear. To address this issue, further study using targeting immunofluorescence on FFD or nano-particles coated FFD is required.

Overall, FFD played an important role in the pathogenesis of disc degeneration by inhibiting cPLA2 activity against TNF-α. Therefore, the FDA-approved FFD may not only be a potential candidate for treating disc degeneration, but also provide an alternative for other TNF-α related diseases.

## Data Availability Statement

The original contributions presented in the study are included in the article/Supplementary Material, further inquiries can be directed to the corresponding authors.

## Ethics Statement

The studies involving human participants were reviewed and approved by The Medical Ethical Committee of Qilu Hospital of Shandong University. The patients/participants provided their written informed consent to participate in this study. The animal study was reviewed and approved by The Professional Committee on Animal Care and Use Committee of Shandong University.

## Author Contributions

JW designed the experiments. KL, GL, and DZ acquired the data. YZ performed the statistical analyses. RL analyzed and interpreted the data. JS maintained the mice. JW, KL, QX, and LC edited the manuscript. All authors drafted and reviewed the manuscript.

## Conflict of Interest

The authors declare that the research was conducted in the absence of any commercial or financial relationships that could be construed as a potential conflict of interest.

## Publisher’s Note

All claims expressed in this article are solely those of the authors and do not necessarily represent those of their affiliated organizations, or those of the publisher, the editors and the reviewers. Any product that may be evaluated in this article, or claim that may be made by its manufacturer, is not guaranteed or endorsed by the publisher.

## Supplementary Material

Supplementary Figure 1 Representative MRI images of the lumbar spine from patients with Pfirrmann grade II (*n* = 40). The right panels show pictures of L5/S1 segments at high magnification.

Supplementary Figure 2 Demonstration for the measurement of central nucleus pulposus height based on H&E staining. Scale bar = 500 μm.
