# Supplementary figures and images for: Fexofenadine Protects Against Intervertebral Disc Degeneration Through TNF Signaling
Source: Front Cell Dev Biol. 2021 Aug 24;9:687024. doi: 10.3389/fcell.2021.687024 (PMC8421647; doi:10.3389/fcell.2021.687024)

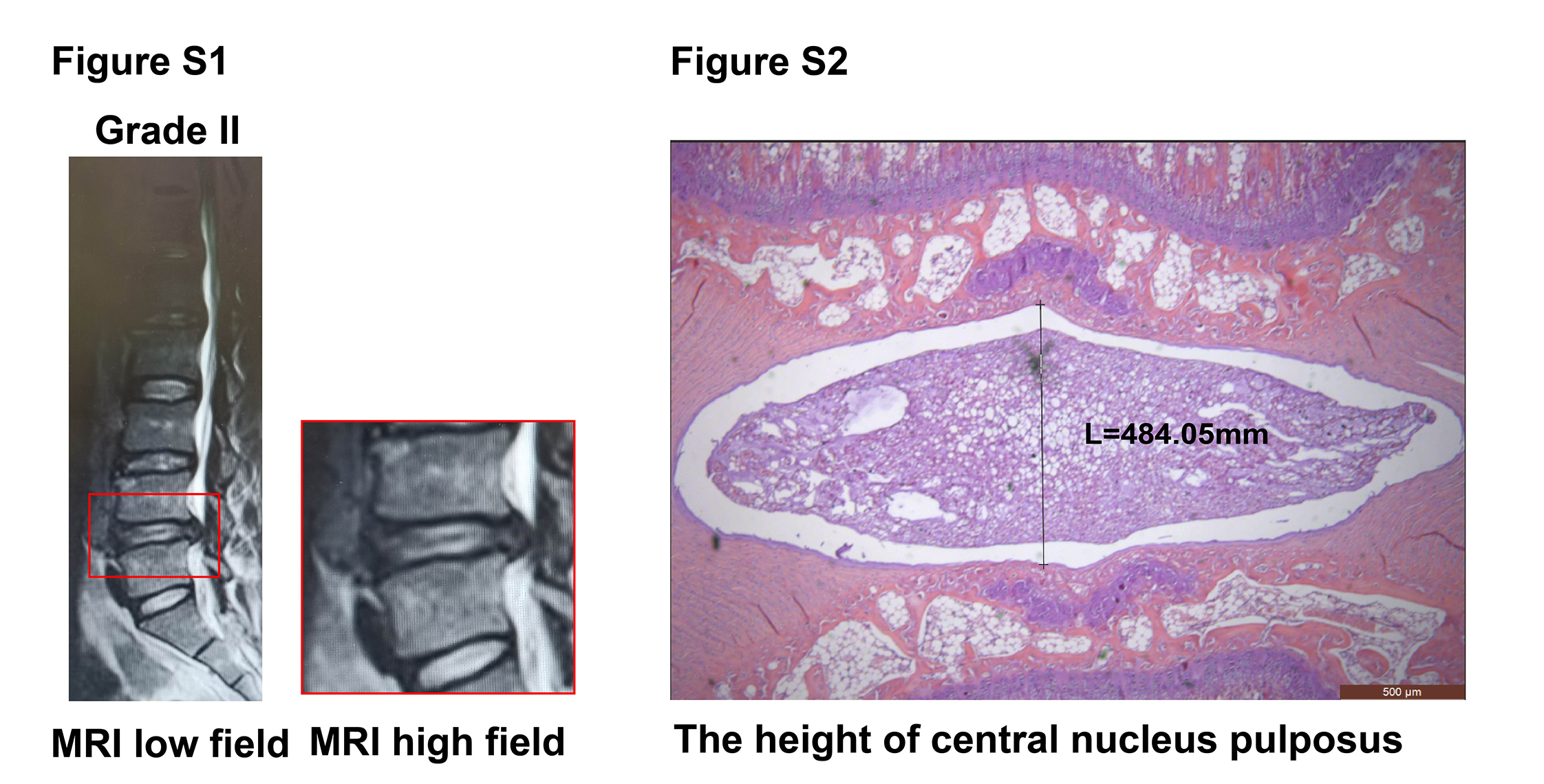

Supplement: Supplementary Figure 1 — Representative MRI images of the lumbar spine from patients with Pfirrmann grade II (n = 40). The right panels show pictures of L5/S1 segments at high magnification. [file Image_1.JPEG]
